# Male Infertility during Antihypertensive Therapy: Are We Addressing Correctly The Problem?

**DOI:** 10.22074/ijfs.2016.4633

**Published:** 2016-09-05

**Authors:** Antonio Simone Laganà, Salvatore Giovanni Vitale, Paola Iaconianni, Simona Gatti, Francesco Padula

**Affiliations:** 1 Unit of Gynecology and Obstetrics, Department of Human Pathology in Adulthood and Childhood “G. Barresi”, University of Messina, Messina, Italy; 2 Department of Reproductive Medicine, Altamedica Fetal Maternal Medical Centre, Rome, Italy; 3 Department of Prenatal Diagnosis, Altamedica Fetal Maternal Medical Centre, Rome, Italy

**Keywords:** Andrology, Infertility, Antihypertensive

## Abstract

Male fertility significantly decreased in the last 50 years, as showed in several studies reporting a reduction of sperm counts per ml in the seminal fluid. Several “acute” pharmacological treatments, as antibiotics, could cause subclinical and temporary reduction of male fertility; conversely, long-term medical treatment may severely affect male fertility, although this effect could be considered transient in most of the cases. Thus, nowadays, several long-term pharmacological treatments may represent a clinical challenge. The association between several kind of antihypertensive drugs and reduction of male fertility has been showed in the mouse model, although the modification(s) which may alter this fine-regulated machinery are still far to be elucidated. Furthermore, well-designed observational studies and randomized controlled trials are needed to accurately define this association in human model, meaning a narrative overview synthesizing the findings of literature retrieved from searches of computerized databases. We strongly solicit future human studies (both observational and randomized clinical trials) on large cohorts with adequate statistical power which may clarify this possible association and the effects (reversible or permanent) of each drug. Furthermore, we suggest a close collaboration between general practitioners, cardiologists, and andrologists in order to choose the most appropriate antihypertensive therapy considering also patient’s reproductive desire and possible risk for his fertility.

## Introduction

Male fertility significantly decreased in the last 50 years, as showed in several studies reporting a reduction of sperm counts per ml in the seminal fluid (1). To date, male factors account for almost 35% of couple infertility. As widely accepted, sperm counts may vary among different ejaculates according to several pathological conditions, lifestyle and exposure to pollutants (2). In this regard, although well-known diseases such as cryptorchidism, varicocele, hypospadias, testicular tumors, Y chromosome microdeletion and endocrine alterations can cause azoospermia and/or oligozoospermia, iatrogenic risk factors may also play a detrimental role in male fertility. Several “acute” pharmacological treatments, as antibiotics, could cause subclinical and temporary reduction of male fertility; conversely, long-term medical treatment may severely affect male fertility, although this effect could be considered transient in most of the cases. Thus, nowadays, several long-term pharmacological treatments may represent a clinical challenge. To the best of our knowledge, association between several kind of antihypertensive drugs and reduction of male fertility has been showed in the mouse model (3,5), although the modification(s) which may alter this fine-regulated machinery are still far to be elucidated and human data are still lacking. Indeed, data from the following animal studies are not robust: several studies (6,8) showed that verapamil, nimodipine, and lisinopril worsen semen quality and testicular morphology, while others (9,10) have found that nifedipine and lisinopril improve these parameters. 

In this regards, Bechara et al. (11) have studied the effects of an angiotensin-converting enzyme (ACE) inhibitor (Enapril) on hypertension-induced morphological changes in the testis and spermatozoid production in spontaneously hypertensive rats. According to their data analysis, sperm concentration was greater in the treated group than in the nontreated group, testicular vascular volumetric density decreased in the nontreated group and, last but not least, volumetric density of the seminiferous epithelium in the treated group was higher than in the nontreated group. Although these results could not be de facto translated in humans, they have suggested a possible pivotal role of ACE inhibitors as first-line treatment when fertility is a relevant concern. Even if well-designed observational studies and randomized controlled trials are needed to accurately define this association in human model, daily clinical experience seems to confirm that in case of antihypertensive therapy with concomitant male infertility, the substitution of the drug with another one which does not affect semen parameters may improve male fertility. 

In particular, beta blockers and calcium-channel blockers (CCBs) seem to play a detrimental role on male fertility, causing in several cases azoospermia and/or oligozoospermia. On the other hand, inhibitors of the funny channel, such as oral ivabradine, seem not to be associated with reduction of male fertility. In this regard, it was already demonstrated in the mouse model that CCBs, like amlodipine, can cause a reduction of testosterone, luteinizing hormone (LH) and follicular stimulating hormone (FSH), leading to affect spermatogenesis and sperm parameters (12,13). However, these data do not seem to be surprising, since accumulating evidence have already suggested that Ca^2+^ plays a prominent role during fertilization in all animal species. On one hand, in mice, rats, pigs, hamsters and bovines, extracellular Ca^2+^ is necessary for epididymal acquisition of sperm motility (14,18). 

Furthermore, it is known to regulate both activated and hyperactivated motility (19,21). In addition, flagellar motility is controlled by calcium through the regulation of dynein-driven microtubule sliding and modulation of the sperm flagellar waveform (22,23). Finally, calcium has a pivotal role during the acrosome reaction in invertebrates, such as echinoderms, and superior vertebrates (24,25). Also, in this case, although these data demonstrated that it does have a direct impact on humans, they may underlie the possible detrimental effect of calcium antagonists administered for hypertension on male fertility. 

## Conclusion

The average life expectancy is increasing and even more older male patients refer to fertility experts, the association between antihypertensive therapy and male infertility is a growing area of interest. Based on this scenario, we strongly solicit future human studies (both observational and randomized clinical trials) on large cohorts and with adequate statistical power which may show this possible association and the effects (reversible or permanent) of each drug. Furthermore, we suggest a close collaboration between general practitioners, cardiologists, and andrologists in order to choose the most appropriate antihypertensive therapy considering also patient’s reproductive desire and possible risk for his fertility. 
